# DivPro: diverse protein sequence design with direct structure recovery guidance

**DOI:** 10.1093/bioinformatics/btaf258

**Published:** 2025-07-15

**Authors:** Xinyi Zhou, Guibao Shen, Yingcong Chen, Guangyong Chen, Pheng Ann Heng

**Affiliations:** Department of Computer Science and Engineering, The Chinese University of Hong Kong, Hong Kong 999077, China; Information Hub, The Hong Kong University of Science and Technology, Guangzhou 511466, China; Information Hub, The Hong Kong University of Science and Technology, Guangzhou 511466, China; Department of Computer Science and Engineering, The Hong Kong University of Science and Technology, Clear Water Bay, Kowloon, Hong Kong 999077, China; Hangzhou Institute of Medicine Chinese Academy of Science, Qiantang District, Hangzhou Zhejiang Province 310000, China; Department of Computer Science and Engineering, The Chinese University of Hong Kong, Hong Kong 999077, China

## Abstract

**Motivation:**

Structure-based protein design is crucial for designing proteins with novel structures and functions, which aims to generate sequences that fold into desired structures. Current deep learning-based methods primarily focus on training and evaluating models using sequence recovery-based metrics. However, this approach overlooks the inherent ambiguity in the relationship between protein sequences and structures. Relying solely on sequence recovery as a training objective limits the models’ ability to produce diverse sequences that maintain similar structures. These limitations become more pronounced when dealing with remote homologous proteins, which share functional and structural similarities despite low-sequence identity.

**Results:**

Here, we present DivPro, a model that learns to design diverse sequences that can fold into similar structures. To improve sequence diversity, instead of learning a single fixed sequence representation for an input structure as in existing methods, DivPro learns a probabilistic sequence space from which diverse sequences could be sampled. We leverage the recent advancements in in silico protein structure prediction. By incorporating structure prediction results as training guidance, DivPro ensures that sequences sampled from this learned space reliably fold into the target structure. We conducted extensive experiments on three sequence design benchmarks and evaluated the structures of designed sequences using structure prediction models including AlphaFold2. Results show that DivPro can maintain high structure recovery while significantly improving the sequence diversity.

**Availability and implementation:**

The source code and datasets are available at https://github.com/veghen/DivPro.

## 1 Introduction

**Figure 1.**
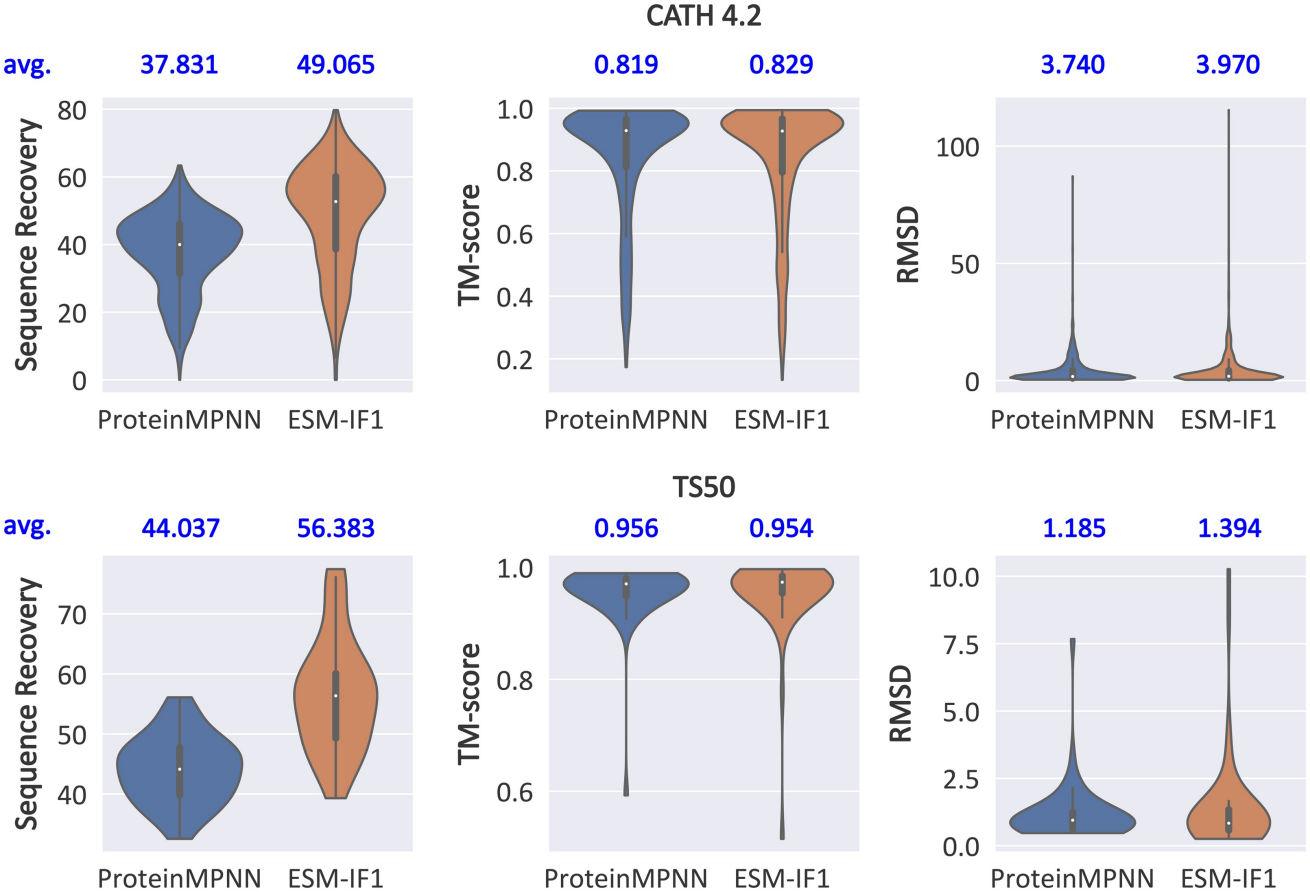
Evaluation of ProteinMPNN and ESM-IF1 on benchmark CATH 4.2 (1120 structures) and TS50 (50 structures), with sequences folded by ESMFold and AlphaFold2 respectively. The inner box plots show the first quartile, median and the third quartile. Whiskers in box plots extend to the most extreme data point that lies within 1.5 times the inter-quartile range (IQR) from the nearest quartile. Mean values are shown above each plot.

The task of Inverse Protein Folding (IPF) is to design protein sequences to achieve specific structures and is essential for various applications, such as *de novo* design of enzymes, biosensors, and therapeutic proteins (Śledź and Caflisch 2018, Pearce and Zhang 2021). Deep learning approaches have demonstrated significant potential in this area, and various strategies have been explored for sequence generation, including one-shot generation (Zhang *et al.* 2020, Anand *et al.* 2022, Gao *et al.* 2023), left-to-right autoregressive generation (Dauparas *et al.* 2022, Hsu *et al.* 2022, Heinzinger *et al.* 2024), generation with gradual refinement (Liu *et al.* 2022, Zhou *et al.* 2023) and denoising with diffusion models (Yi *et al.* 2023). Many existing methods have adopted native sequence recovery as both their training objective and evaluation metric. These approaches generate sequences from protein backbone structures and optimize the model to minimize discrepancies between the generated and native sequences. This is motivated by the assumption that similar sequences will adopt similar conformations (Rost 1999, Kosloff and Kolodny 2008, Chakravarty *et al.* 2023). However, recent studies have revealed an increasing number of inconsistencies between sequence and structural similarities. On one hand, highly similar sequences with only a few mutations can fold into distinct structures (Kosloff and Kolodny 2008, Kim *et al.* 2021, Chakravarty *et al.* 2023). On the other hand, remote homologous proteins with highly similar topologies often exhibit low-sequence similarity (Zou and Saven 2000, Krissinel 2007). This is further supported by our experimental observations on two classic IPF models, ProteinMPNN (Dauparas *et al.* 2022) and ESM-IF1 (Hsu *et al.* 2022), on two popular benchmarks CATH 4.2 (Ingraham *et al.* 2019) and TS50 (O’Connell *et al.* 2018). As shown in Fig. 1, we evaluate their sequence recovery, which measures the percentage of identical residues between generated and wild-type (WT) sequences. We fold the generated sequences and compare the predicted structures with WT structures to measure structure recovery metrics, Template Modeling (TM) score (Zhang and Skolnick 2004), and Root Mean Square Deviation (RMSD). We use ESMFold (Lin *et al.* 2023) to fold sequences from CATH 4.2 and AlphaFold2 (Jumper *et al.* 2021) for TS50. ESM-IF1 achieves significantly higher sequence recovery but does not show corresponding improvements in structure recovery. These results indicate the importance of learning the complex sequence–structure relationship to identify structure-preserving mutations. Otherwise, structural conservation may not be guaranteed even when sequence similarity is high.

Another limitation of current IPF methods is that they do not explicitly address sequence diversity in their design objectives. Designing diverse amino acid sequences that share similar structures holds significant value for both research and applications. Theoretically, it can enhance our understanding of protein structure robustness and evolutionary adaptability (Murphy *et al.* 2012). Furthermore, sequence variations that maintain structural similarity can yield novel functionalities and broaden the candidate pool, benefiting downstream tasks including drug design and therapeutics (Dellas *et al.* 2021, Repecka *et al.* 2021, Ebrahimi and Samanta 2023). However, most existing methods are constrained by their architecture: they learn only a single fixed sequence representation for each input structure and optimize primarily for sequence recovery during training. While some sequence diversity can be achieved through probability sampling from the final distribution prediction of amino acid types, this approach remains limited in its scope (Dauparas *et al.* 2022, Hsu *et al.* 2022).

To address the above limitations, we present DivPro, a model designed to generate diverse sequences for a target protein structure. Instead of learning a single deterministic sequence representation for a structure, our model learns a probabilistic sequence representation space. This approach enables sampling of diverse sequences from this representation space, similar to the methodology employed in variational autoencoder (VAE) (Kingma and Welling 2013). To ensure the sampled sequences can preserve the target structure, we incorporate explicit structure-based guidance during training. We show that the model is able to learn a biologically meaningful sequence representation space that captures structural relationships. Experiment results demonstrate that DivPro greatly improves generated sequence diversity compared to the state-of-the-art (SOTA) models while maintaining high structure recovery performance.

## 2 Related works

IPF is the task to find amino acid sequences that can fold into a given 3D protein backbone structure. It has been an important protein design problem for decades (Ingraham *et al.* 2019). Here, we focus on deep learning studies related to this task. Early works in this field represent protein structures as hand-crafted features and use simple feed-forward networks to predict amino acids independently (Li *et al.* 2014). Some methods represent proteins as atom point clouds, where structural features are encoded by atomic coordinates. These approaches employ 3D convolutional neural networks to process the structural features and generate protein sequences (Zhang *et al.* 2020, Anand *et al.* 2022). Many recent studies model protein structure as graphs, with amino acids as graph nodes and their interaction as graph edges. ProteinMPNN (Dauparas *et al.* 2022) computes interatomic distances as edge features and employs a message passing graph neural network to decode protein sequences autoregressively. ESM-IF1 (Hsu *et al.* 2022) learns protein features through Geometric Vector Perceptron layers (Jing *et al.* 2021) and uses a transformer architecture (Vaswani 2017) for sequence generation. They augment the training data with AlphaFold2 predicted structures, which significantly improves the model’s sequence recovery. PiFold (Gao *et al.* 2023) achieves high-sequence recovery and inference speed by carefully designed residue features and network architecture to learn the extracted features. Yi *et al.* (2023) developed a graph diffusion model that gradually refines random amino acid sequences conditioned on structural information. Their approach employs an equivariant graph neural network as the denoising mechanism. Recently, protein language models have demonstrated great success across various downstream tasks, including sequence design and structure prediction (Lin *et al.* 2023, Madani *et al.* 2023). A recent work by Qiu *et al.* (2024) proposed InstructPLM, which tackles IPF problem by prompting pretrained protein language models with learned structure instructions. It achieves new SOTA sequence recovery on the CATH 4.2 dataset.

## 3 Methods

**Figure 2.**
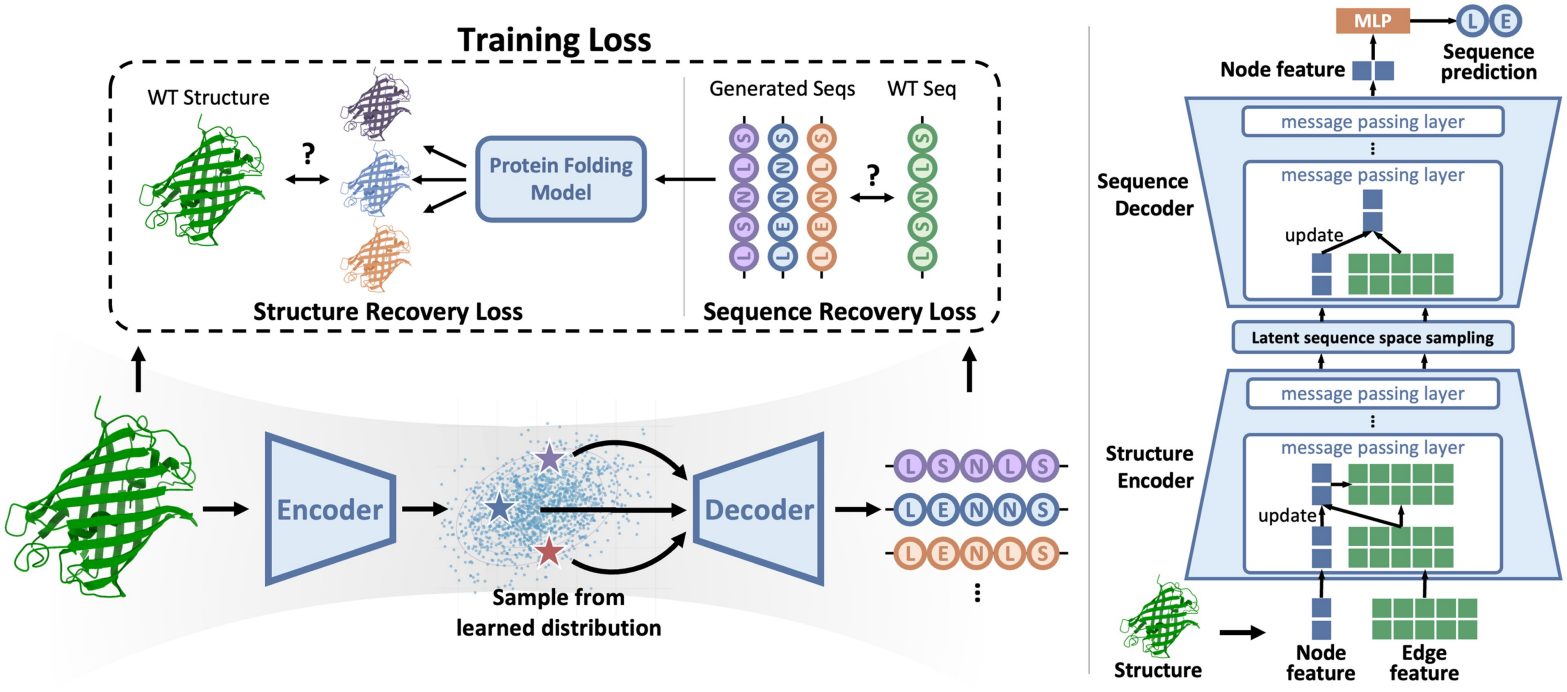
Left: The overview of our method. A protein structure is encoded into a latent sequence space, where the sampled sequences can fold into similar structures as the target structure. A pretrained protein folding model is employed to provide structure recovery guidance during training to learn a plausible sequence space. Right: DivPro model architecture. The encoder and decoder employ message passing layers to update node and edge features. The final node features are fed into an MLP to predict the sequence.

In this section, we first provide the preliminaries of encoder-decoder-based sequence generation models. Then we will introduce our DivPro implementation and training methodology. The overview of our method is illustrated in Fig. 2.

### 3.1 Preliminary: encoder–decoder-based sequence generation from structures

Encoder–decoder architectures are widely adopted in sequence generation tasks. The architecture can be generalized as follows: Given an input protein backbone structure *S* with *N* residues, the sequence prediction is obtained from p=Dec(Enc(S)), where $p\in R^{N\times 20}$ is the probability distribution over 20 amino acid types for each residue. The structure encoder Enc transforms *S* into sequence representations using various architectures such as message passing layers (Jing *et al.* 2021, Dauparas *et al.* 2022), transformer encoder (Hsu *et al.* 2022) or graph attention layers (Gao *et al.* 2023). The decoder Dec decodes sequence representations into probability distributions, which can be the same architecture as Enc (Dauparas *et al.* 2022, Hsu *et al.* 2022) or simple MLP layers (Gao *et al.* 2023).

In existing methods, the encoder Enc is typically deterministic, producing a fixed sequence representation Enc(S) for a given input structure *S*. To generate diverse sequences, randomness is introduced either by sampling from the predicted probability distribution p or by randomizing the decoding order in Dec (Dauparas *et al.* 2022). However, these methods do not directly address the essential many-to-one mapping problem. Inspired by VAEs, we propose to design a model that learns Enc(S) as a distribution and different sequences can be sampled from the learned distribution space.

### 3.2 Protein structure representation

We represent a protein backbone structure *S* as a k-nearest-neighbor graph with k=48. Each residue is modeled as a graph node. An edge is constructed between a residue node and its *k*-nearest neighbors in terms of $C_{\alpha }$ distance. We denote the constructed graph as G=(V,E), where V is the residue node set and E is the edge set. Following Dauparas *et al.* (2022), we extract interatomic distances between backbone atoms $N,C_{\alpha },C,O$ and a virtual $C_{\beta }$ and pass the extracted distances through linear layers to obtain the edge features. We use zero features for node features. The resulting features are denoted as node features $H\in R^{N\times d}$ and edge features $E\in R^{N\times k\times d}$, where *N* is the number of residues and *d* is the feature dimension. We use $H_{i}$ to denote the feature vector of node *i* and $E_{\mathrm{ij}}$ for the feature vector of the edge between node *i* and *j*.

### 3.3 DivPro model architecture

#### 3.3.1 Protein structure encoding

A structure encoder composed of several encoder layers first encodes the features H and E into latent sequence space. In each layer, the node features will be updated and then be used to update the edge features. Suppose $H^{l}$ and $E^{l}$ are the inputs to the *l*th encoder layer. We employ the message passing operation (Gilmer *et al.* 2017) for the feature update in each layer:


(1) $$H_{i}{}^{l+1}=H_{i}{}^{l}+\frac{1}{k}\sum_{j\in N_{i}}\;\text{Linear}(H_{i}{}^{l},E_{\mathrm{ij}}{}^{l},H_{j}{}^{l}),$$

(2) $$E_{\mathrm{ij}}{}^{l+1}=E_{\mathrm{ij}}{}^{l}+\text{Linear}(H_{i}{}^{l+1},E_{\mathrm{ij}}{}^{l},H_{j}{}^{l+1}),$$

where $N_{i}$ is the set of *k* neighbors of node *i* and $H_{i}{}^{l+1},E_{\mathrm{ij}}{}^{l+1}$ are the updated features. For clarity of presentation, we omit the activation functions and normalization layers. After several layers of feature update, the final node and edge features produced by the encoder are denoted as $H^{\text{Enc}}$ and $E^{\text{Enc}}$.

#### 3.3.2 Latent sequence space learning

To capture the ambiguity of the sequence-to-structure mapping, we model each sequence representation as a multivariate Gaussian distribution in the latent space. The latent distribution is parameterized by mean $\mu \in R^{N\times d}$ and standard deviation $\sigma \in R^{N\times d}$, which are obtained through a linear transformation from the node features $H^{\mathrm{Enc}}$ produced by the encoder:


(3) $$\mu ,\sigma =\text{Linear}(H^{\mathrm{Enc}}).$$

Then different sequence embeddings could be sampled from the learned distribution. To enable gradient backpropagation, we apply the reparameterization trick to sample the latent sequence embedding $Z\in R^{N\times d}$:


(4) Z=μ+σ⊙ϵ, ϵ∼N(0,I),

where ϵ is sampled from a standard normal distribution and ⊙ denotes element-wise multiplication.

#### 3.3.3 Protein sequence decoding

The sequence decoder Dec is then used to decode the output sequence based on the sampled latent embedding Z and the edge features $E^{\text{Enc}}$. With a little abuse of notation, we denote the input node features to the *l*th decoder layer as $H^{l}$, with $H^{0}=Z$. Each decoder layer updates the node features according to:


(5) $$H_{i}{}^{l+1}=H_{i}{}^{l}+\frac{1}{k}\sum_{j\in N_{i}}\text{Linear}(H_{i}{}^{l},E_{\mathrm{ij}}^{\mathrm{Enc}},H_{j}{}^{l}).$$

Our model generates sequence autoregressively and previously generated residue information is concatenated with the node features. Finally, the node features from the last layer of decoder $H^{\mathrm{Dec}}$ are used to predict the sequence probability $p\in R^{N\times 20}$:


(6) $$p=\text{Softmax}(\text{Linear}(H^{\mathrm{Dec}})).$$

### 3.4 Structure guidance

To guide the model to learn a latent sequence space that can reconstruct the target protein structure, we introduce a structure recovery loss. We employ a single-sequence protein structure prediction model trRosettaX-Single (Wang *et al.* 2022), which is end-to-end differentiable and therefore suitable for serving as a training guidance. The folding model, denoted as F, folds the predicted sequence s to a reconstructed structure $S^{r}$. The predicted structure $S^{r}\in R^{N\times N\times b}$ is represented as a distance map of the distances between $C_{\beta }$ of all residues within the protein structure. The continuous distance values are discretized into *b* bins where each bin corresponds to a specific range of distances, converting the problem into a classification task. Therefore, a cross-entropy loss function is employed to quantify the accuracy of the reconstructed structure with respect to the native structure:


(7) $$L_{\mathrm{struct}}=\text{CrossEntropy}(S^{r},\text{DistanceMap}(S)),$$

(8) $$S^{r}=F(s),$$

where DistanceMap is the function that calculates the distance map from the native structure *S*. One of the advantages of using distance maps to represent protein structures is their invariance to transformations such as translation and rotation, which obviates the need for prior alignment of the structures.

To obtain discrete sequence s from probability prediction p during training, one cannot use the argmax operation, which is an undifferentiable operation preventing the propagation of gradients. To address this problem, we adopt the Gumbel-Softmax (Jang *et al.* 2016) to allow gradients to propagate:


(9) $$s=s_{\mathrm{hard}}-\text{Detach}(p)+p,$$

(10) $$s_{\mathrm{hard}}=\text{argmax}(p).$$

The structure recovery loss $L_{\text{struct}}$ guides the model to learn a latent sequence space, in specific, the parameters μ and σ, where the decoded sequences would adopt structures similar to input ones.

### 3.5 DivPro training

#### 3.5.1 Loss functions

Besides structure recovery loss $L_{\text{struct}}$, we also compute the traditional negative log-likelihood loss over p as the sequence recovery loss, which we found helpful for stabilizing the training process. We denote this loss as $L_{\text{seq}}$.

Additionally, we adopt the Kullback–Leibler divergence loss from VAE to regularize the spread σ of the distribution, which also enhances training stability:


(11) $$L_{\sigma }=\frac{1}{2}\sum_{i}\left(\sigma_{i}^{2}-\text{log}\;\sigma_{i}^{2}-1\right).$$

The total loss is the weighted sum of the three loss functions:


(12) $$L=w_{1}\cdot L_{\mathrm{seq}}+w_{2}\cdot L_{\mathrm{struct}}+w_{3}\cdot L_{\sigma }.$$

Empirically, we set $w_{1}=1,w_{2}=2,w_{3}=0.01$.

#### 3.5.2 Two-stage training strategy

DivPro is trained on the CATH 4.2 training set with a two-stage training strategy. In the first stage, the model is trained on all 18024 structures using only sequence recovery loss. In the second stage, we include the structure recovery loss $L_{\text{struct}}$ and regularization loss $L_{\sigma }$ as discussed above. To ensure reliable and accurate structure recovery guidance, training on the second stage is restricted to 4126 complete structures in CATH 4.2 training set which do not contain missing regions. Furthermore, the structural recovery loss $L_{\text{struct}}$ is applied only to sequences with sequence recovery scores above 0.2. There are two main reasons for adopting a two-stage training strategy. First, it improves training efficiency by allowing the model to initially learn a reasonable latent sequence space using only sequence-level loss, which is less computationally demanding than structure recovery loss. In the second stage, the model can focus on refining the latent sequence space under the guidance of the structure recovery loss. Second, it ensures training stability by avoiding noisy and uninformative feedback from the structure prediction model during the early stages, when the model’s predictions are poor and often produce unnatural sequences. Therefore, the first stage provides a more stable and meaningful starting point for the second stage. We perform validation on the CATH 4.2 validation set for both stages.

#### 3.5.3 Implementation and training

We use a feature size of d=128. The structure encoder and sequence decoder each consist of 3 layers. We employ Gaussian Error Linear Units as activation functions and apply layer normalization following linear transformations in both encoder and decoder. Residual connections are employed in each layer to facilitate gradient flow during training. In the first training stage, we train the model for 150 epochs using a batch size of 16, a learning rate of $1\times 10^{-4}$, and a dropout rate of 0.1. For the second training stage, we continue training for an additional 20 epochs with a batch size of 3 and a lower learning rate of $3\times 10^{-5}$, while maintaining the same dropout rate. To enhance computational efficiency and reduce memory usage, we implement mixed-precision training during this second stage. For both training stages, we use the Adam optimizer and adjust the learning rate during training process using cosine annealing schedule.

## 4 Results

### 4.1 Structure recovery and sequence diversity

We evaluate the model’s ability to recover the input target structures and the diversity of generated sequences.

#### 4.1.1 Datasets

We evaluate on three popular IPF benchmarks: CATH 4.2 (Ingraham *et al.* 2019) test split (*n* = 1120), TS50 (*n* = 50), and TS500 (*n* = 470) (O’Connell *et al.* 2018).

#### 4.1.2 Baselines

We first construct two heuristic methods for improving sequence diversity: RandMut method which mutates residues on native sequences randomly into other amino acids, and BLOSMut which mutates residues using normalized BLOSUM62 amino acid substation matrix (Henikoff and Henikoff 1992) as transition probability. We set the mutation rate to 0.25 for both methods, meaning that each residue has a 25% probability of being mutated. These two heuristic methods are constructed to investigate how these controlled perturbations to native sequences affect structure preservation.

We further compare DivPro with recent competitive Inverse Folding models: ProteinMPNN (Dauparas *et al.* 2022) (48 edges, 0.20 Å noise version), ESM-IF1 (Hsu *et al.* 2022), PiFold (Gao *et al.* 2023), and InstructPLM (Qiu *et al.* 2024). While PiFold and InstructPLM are trained on the same training set as our method, ProteinMPNN is trained on a custom PDB structure dataset with 25 361 structure clusters and ESM-IF1 is trained on PDB structures augmented with 12 million AlphaFold2 predicted structures. Note that the original PiFold model does not produce predictions for residues on missing regions, resulting in generated sequences that are incomplete and contain gaps. To ensure that the generated sequences are foldable by structure prediction models, we have adjusted PiFold to predict for missing residues similar to the other models.

#### 4.1.3 Experiment settings

For each structure, 20 sequences are sampled by each method and folded by protein structure prediction models. We make sure that the structure prediction models used for evaluation are different from the ones used for training (trRosettaX-Single) to prevent overfitting on a certain folding algorithm. From the 20 generated sequences, the top 5 with the highest TM scores against the native structure are selected for evaluation on the following metrics:

- TM score: TM score between the native structure and the predicted structure. Higher values indicate better alignment.
- RMSD: Root Mean Square Deviation between the native structure and the predicted structure. Lower values indicate better alignment.
- Diversity: The complement of the average pairwise sequence similarity across the five generated sequences. Higher scores indicate a more diverse generation.

#### 4.1.4 Results evaluated by ESMFold

**Figure 3.**
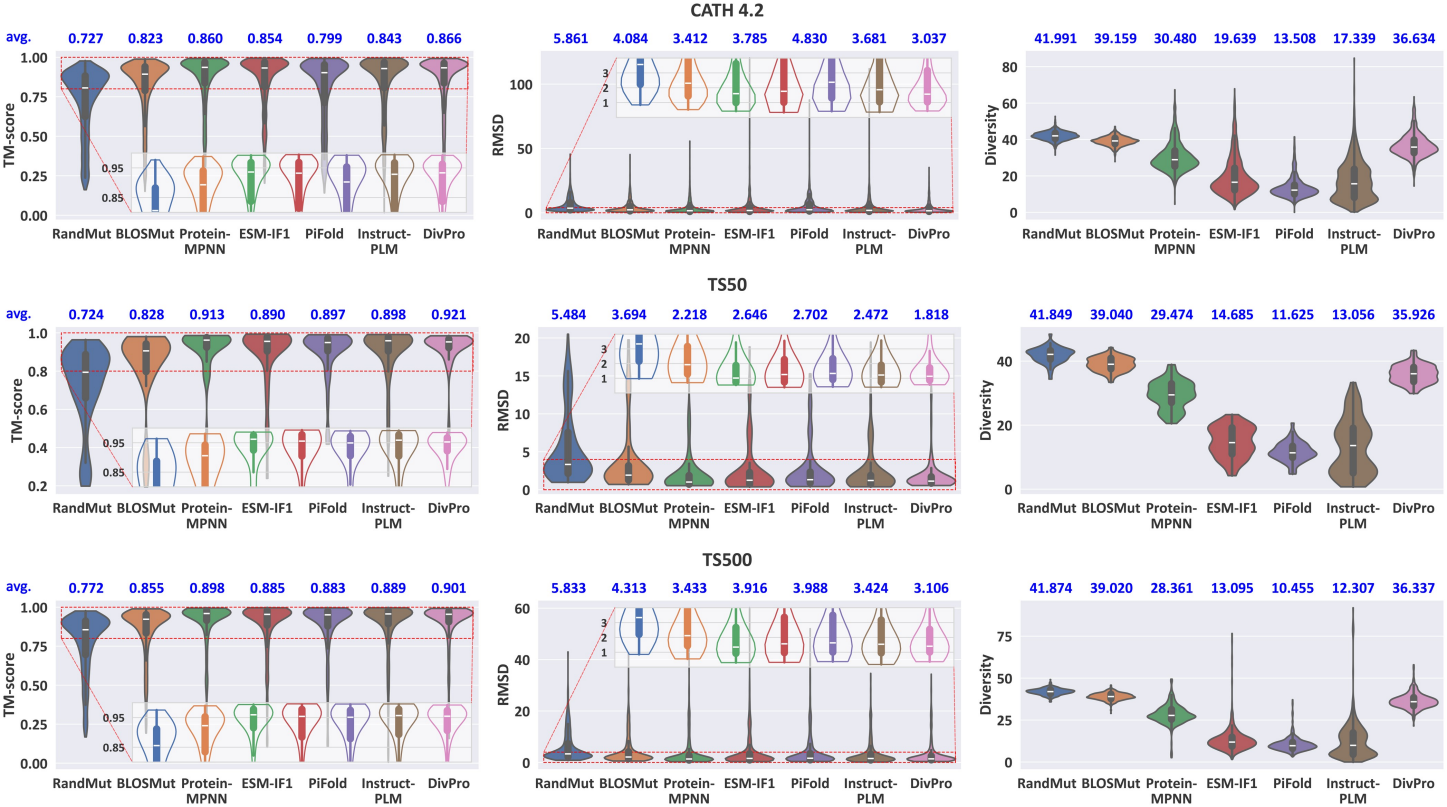
Performance evaluated by ESMFold on three benchmarks. Box plots show the quartiles (Q1, median, Q3) with whiskers extending to data points within 1.5 IQR of the quartiles. Mean values are shown above each plot. Dashed boxes in the Template Modeling (TM) score and RMSD plots highlight regions shown in corresponding inset plots, showing core distributions without extreme outliers.

We first evaluate the generated sequences by ESMFold (Lin *et al.* 2023), which provides efficient and accurate structure prediction. Results are shown in Fig. 3. While the heuristic baselines (RandMut and BLOSMut) achieve high-sequence diversity due to their high mutation rate, they show poorer structure recovery compared to other methods, as evidenced by their lower TM scores and higher RMSD values. BLOSMut performs better than RandMut in structure recovery, suggesting that BLOSUM62-guided mutations produce more structurally stable sequences. Notably, DivPro achieves sequence diversity comparable to the two heuristic baselines while better preserving the target structures, shown by its higher TM scores and lower RMSD values across all benchmarks. These results indicate that our model has learned to select mutations that effectively preserve the original structures.

Furthermore, DivPro outperforms the deep learning baselines. It achieves better structure recovery (higher average TM scores and lower RMSD values) and also obtains significantly higher sequence diversity than ESM-IF1, PiFold, and InstructPLM. While matching the structure recovery performance of the best baseline model, ProteinMPNN, DivPro improves the average sequence diversity by at least 20%.

#### 4.1.5 Results evaluated by AlphaFold2

**Figure 4.**
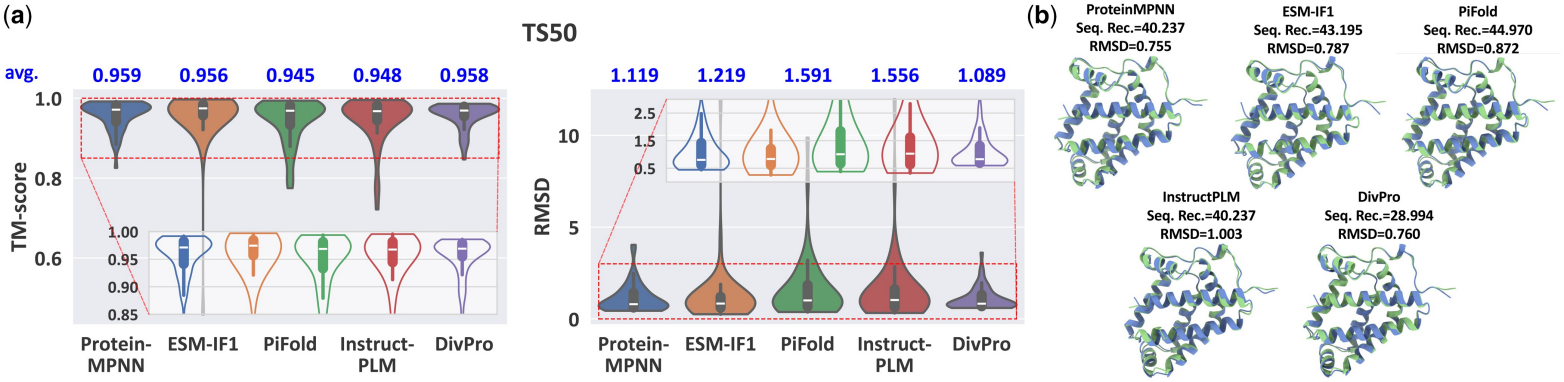
(a) Performance evaluated by AlphaFold2 on TS50 benchmark. Box plots show the quartiles (Q1, median, Q3) with whiskers extending to data points within 1.5 IQR of the quartiles. Mean values are shown above each plot. Dashed boxes highlight regions shown in corresponding inset plots, showing core distributions without extreme outliers. (b) The AlphaFold2 predicted structures of generated sequences for an example protein 1OR4. Predicted structures are shown in blue while native structures are shown in green.

To further validate our results, we evaluate the sequences by AlphaFold2, currently recognized as the state-of-the-art protein structure prediction model. Due to limited computing resources, we only evaluate on the TS50 dataset, and for each structure, we randomly select one sequence from the top five sequences when evaluated by ESMFold. Results are presented in Fig. 4a. Since one sequence is sampled for each structure, the diversity metric is not calculated. Similar to results obtained from ESMFold, ProteinMPNN demonstrates better ability to reconstruct the target structures than other baseline models. Our model achieves comparable performance on both TM score and RMSD metrics. Figure 4b presents a representative case selected from the TS50 dataset (PDB code 1OR4). The sequences designed by models are folded via AlphaFold2. The predicted structures are shown in blue, and native structures are shown in green. The sequence designed by DivPro is highly diverse from the native one compared to other models (sequence recovery rate below 30%). However, it still folds into the structure that closely matches the native conformation with low RMSD.

#### 4.1.6 Sequence quality analysis

**Figure 5.**
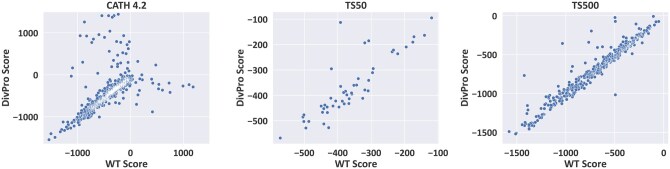
Comparison of Rosetta energy scores of native structures and corresponding structures predicted from DivPro-designed sequences.

Additionally, to assess the quality of generated sequences, we evaluate the energy scores of both WT structures and predicted structures of generated sequences. We employ the minimize.static.linuxgccrelease Rosetta protocol (Das and Baker 2008) to minimize the structures and calculate the Rosetta scores. Lower Rosetta scores indicate more energetically favorable conformations according to Rosetta’s energy function. The score distributions are visualized in Fig. 5. Across all three test datasets, we observe similar scores between WT structures and their corresponding DivPro-designed structures for the majority of proteins. These results demonstrate DivPro’s ability to generate high-quality protein sequences that yield energetically favorable structures comparable to those of native proteins.

### 4.2 Analysis of learned sequence feature space

To investigate whether our model’s learned sequence feature space captures structural relationships between proteins, we conduct an analysis using 47 proteins with varying degrees of structural similarity. These proteins are obtained by performing a structure-based search using the Foldseek server (Van Kempen *et al.* 2024) with a query structure (PDB 6K80). The selected proteins exhibit a wide range of structural similarities, with TM scores ranging from 0.22 to 0.99 relative to the query structure.

**Figure 6.**
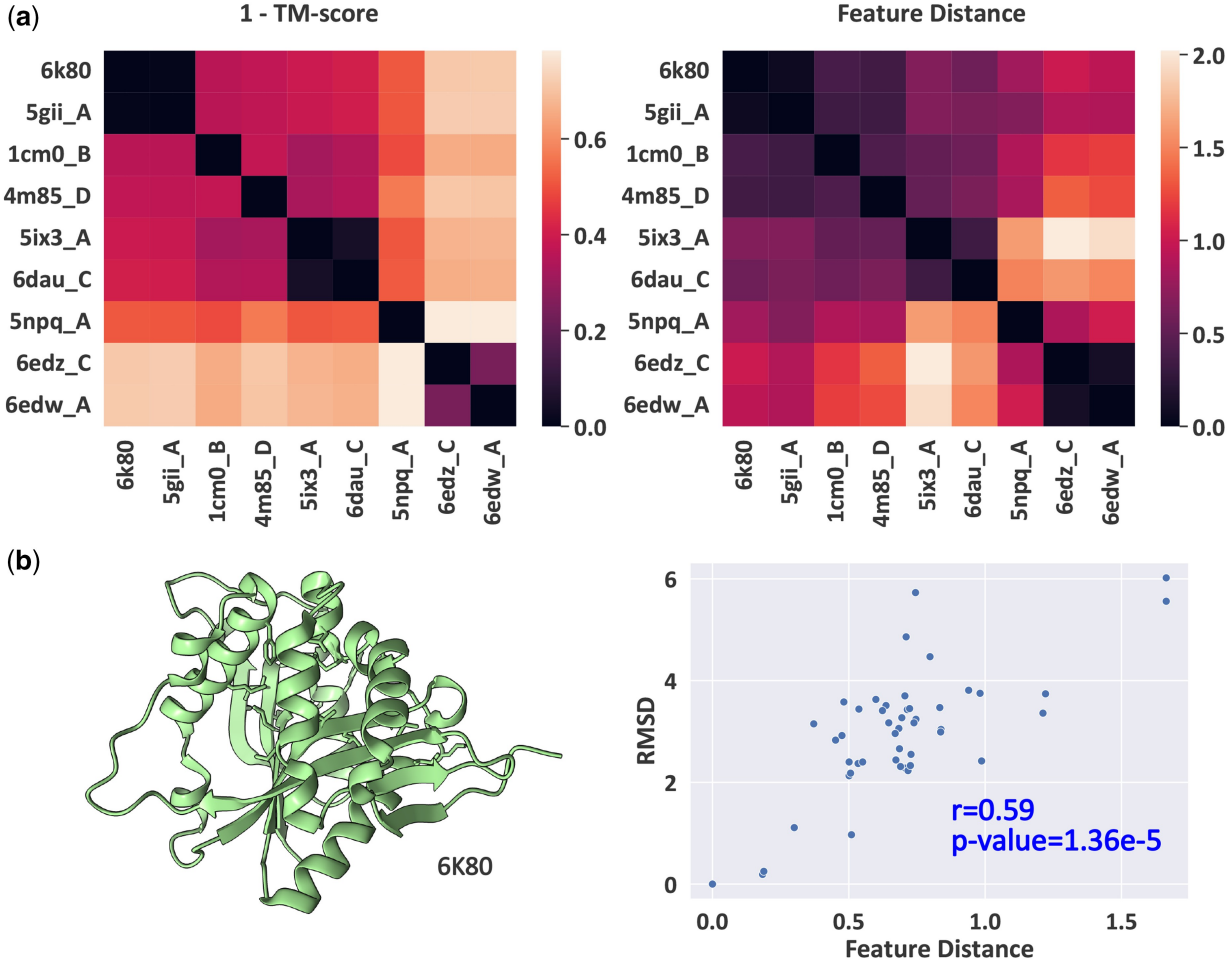
(a) Comparison of structural distances and learned feature distances. Left: structure dissimilarity matrix (1 − Template Modeling (TM) score), where higher values indicate more dissimilar structures. Right: Pairwise distances in DivPro’s learned feature space. (b) Left: structure of query protein 6K80. Right: Scatter plot showing the relationship between RMSD and feature space distances from protein 6K80. Each point represents a protein.

For each protein, we generate 20 sequences using DivPro and compute the mean sequence features to obtain a representative point in the feature space. Then we compute the pairwise Euclidean distance between these feature points to obtain the feature distance matrix. We perform Mantel tests (Mantel 1967) between the feature distance matrix and two structure similarity metrics: 1 − TM score and RMSD. We take the complement of TM score to ensure higher values indicate more dissimilar structures in all three matrices. The feature distances show significant correlation with both 1 − TM score (*r* = 0.40, *P* < .001) and RMSD (*r* = 0.59, *P* < .0001). These correlations demonstrate that our model has learned to encode structural relationships and similarities in its latent sequence space, so that the sampled sequences can preserve the target protein structure. In Fig. 6a, we visualize the 1 − TM score matrix and feature distance matrix of a subset of proteins for visualization clarity. The heat maps of two matrices demonstrate similar patterns.

Additionally, we perform a focused analysis on the query protein (PDB 6K80). We examine how the distances from 6K80 to other proteins in the feature space correlate with their actual structural differences. Using Pearson correlation analysis, we observe that the feature space distances strongly correlate with both 1 − TM score (*r* = 0.47, *P* < .001) and RMSD (*r* = 0.59, *P* < .0001). These results indicate that proteins that are structurally similar to 6K80 are mapped closer to it in the latent feature space, while structurally distinct proteins are mapped farther away. This further supports that the learned feature space by DivPro effectively captures structural relationships. We plot the scatter plot between RMSD and feature space distances from protein 6K80 in Fig. 6b.

### 4.3 Application: protein complex design

**Figure 7.**
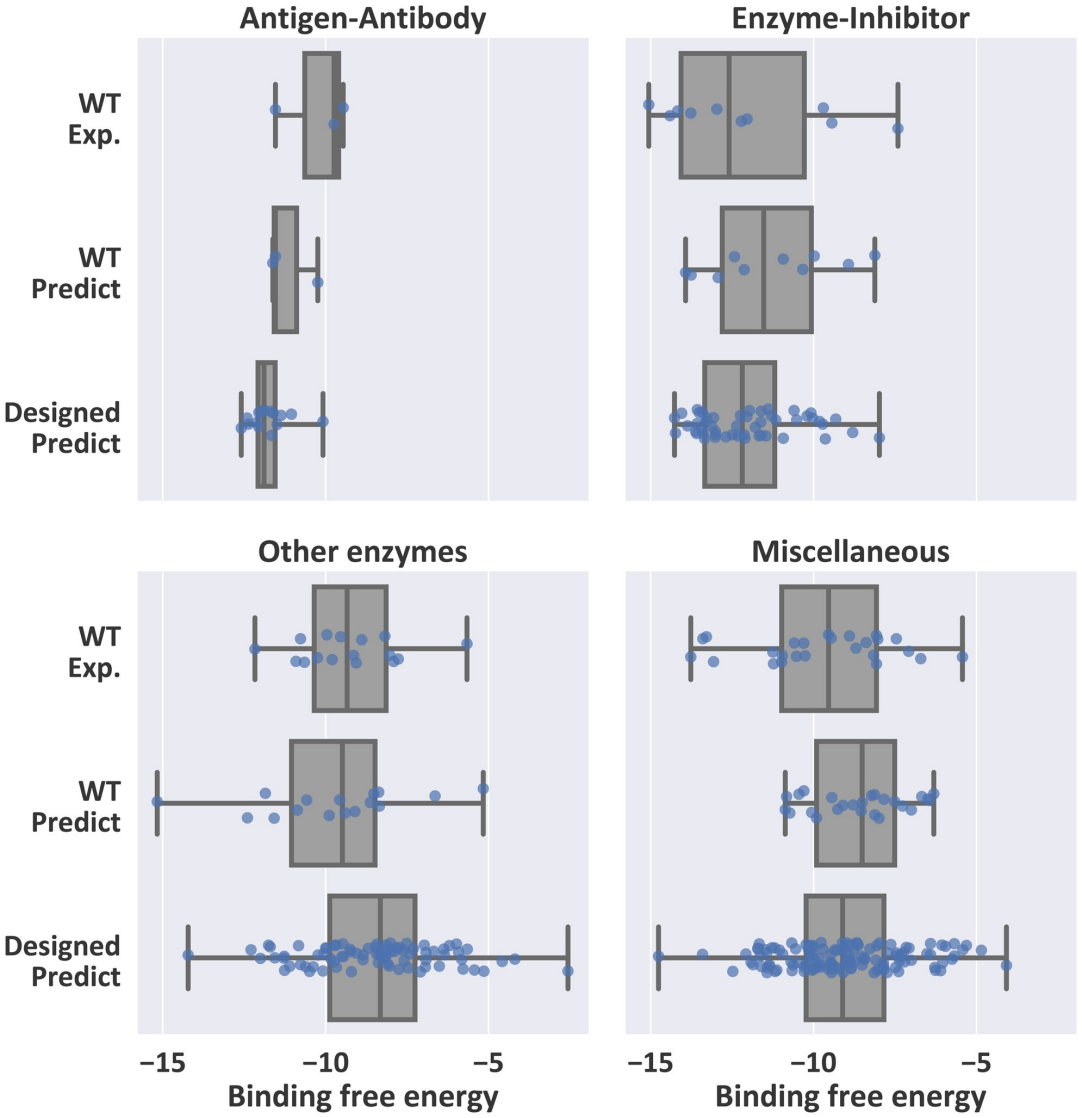
The distribution comparison of the experimental binding affinities of WT complexes, predicted affinities of WT complexes and the predicted affinities after redesigning one of the protein chains by DivPro.

To further evaluate DivPro’s ability to preserve functional properties while designing protein sequences, we apply our method to the task of protein complex redesign. We take 54 protein complexes from the dataset in Liu *et al.* (2015). For each complex, we randomly choose one protein chain and use DivPro to generate five sequences based on the structure of the selected chain. To assess whether these redesigned sequences retain their binding functionality, we predict the binding affinity of the redesigned complexes using the binding affinity prediction server PPA-Pred2 (Nikam *et al.* 2018). In Fig. 7, we provide the comparison of experimentally determined affinities of the wild-type complexes, PPA-Pred2 predicted affinities for the WT complexes, and predicted affinities for the complexes containing our redesigned sequences. By including both experimental and predicted affinities for WT complexes, we can assess the accuracy of PPA-Pred2’s predictions and also provide a more reliable baseline to evaluate our generated sequences. Notably, the redesigned complexes maintain comparable binding affinities to the WT complexes across most complex function classes, with some redesigned variants even exhibiting enhanced predicted binding strength. These results demonstrate DivPro’s ability to generate sequences that can fold into the desired structure and also preserve critical functional properties.

## 5 Discussion and conclusion

Our work presents DivPro, a model for protein sequence design that explicitly models the inherent diversity in sequence–structure relationships. DivPro models sequence representations as probabilistic distributions rather than deterministic point estimates and learns this distribution under structure guidance. The two key innovations in DivPro, i.e. probabilistic sequence representation learning and structure recovery objective, work together to balance sequence diversity with target structure preserving. The latent sequence space sampling enhances the generation of diverse sequences, while the structure recovery guidance constraints this diversity to a viable sequence space. Our method performs better in generating diverse sequences that preserve the input structure and protein functionality. The strong correlation between structural and feature space distances suggests that our learned representations effectively preserve the protein structure information.

There are several limitations of this study. First, as discussed in Section 3.5, training with structure recovery loss involves a complex structure prediction model in the gradient calculation and backpropagation, making it computationally expensive and GPU memory-intensive. While we mitigate this issue through a two-stage training strategy, this approach still imposes constraints on the size of the dataset and the model architecture that can be used. Future work could focus on developing more efficient methods for providing structure recovery guidance, such as alternative loss formulations, approximation techniques (Melnyk *et al.* 2022), lightweight surrogate models (Hamamsy *et al.* 2024) or preference learning using reinforcement learning (Ouyang *et al.* 2022). This would allow training on larger datasets and exploring more complex model architectures, which could further improve performance and generalization.

Another limitation of this study is that the structure recovery metrics are only assessed by in silico structure prediction algorithms, due to the high cost of experimentally solving protein structures on large-scale datasets. Nonetheless, the folding algorithms used in the evaluation are generally considered highly accurate and are widely adopted as in silico validation tools by many existing works (Dauparas *et al.* 2022, Watson *et al.* 2023, Mifsud *et al.* 2024). To mitigate this issue and provide a more comprehensive evaluation, we incorporate additional evaluation methods, such as Rosetta and PPA-Pred2, to complement structure prediction models by providing evaluations from different perspectives. Thus, the results presented can still provide valuable insight and guide future research in the field of structure-based protein design.

## Author contributions

Xinyi Zhou (Software [lead], Data curation [lead], Formal analysis [lead], Investigation [lead], Methodology [supporting], Visualization [lead], Writing--original draft [lead], Writing--review & editing [lead]), Guibao Shen (Conceptualization [lead], Methodology [lead], Software [supporting], Writing--original draft [supporting], Writing--review & editing [supporting]), Yingcong Chen (Supervision [equal], Validation [equal], Writing--review & editing [supporting]), Guangyong Chen (Supervision [equal], Funding acquisition [equal], Validation [equal], Writing--review & editing [supporting]), and Pheng Ann Heng (Supervision [equal], Funding acquisition [equal], Validation [equal], Writing--review & editing [supporting])

Conflict of interest: None declared.

## Funding

This work was supported by funds from the National Natural Science Foundation of China (#62376254 and #32341018) and Hong Kong Innovation and Technology Fund (Project no. ITS/241/21).

## Data Availability

DivPro, TS50 dataset and TS500 dataset are available at https://github.com/veghen/DivPro. The CATH 4.2 dataset is available at https://people.csail.mit.edu/ingraham/graph-protein-design/data/. The protein complex dataset is available at https://www.iitm.ac.in/bioinfo/PPA_Pred/index.html.
